# Social phobia of Ethiopian students: meta-analysis and systematic review

**DOI:** 10.1186/s13643-023-02208-2

**Published:** 2023-03-14

**Authors:** Mamaru Melkam, Tesfaye Segon, Girum Nakie

**Affiliations:** Department of Psychiatry, College of Medicine and Health Sciences, University of Gondar, Gondar, Ethiopia

**Keywords:** Social anxiety disorder, Social phobia, Meta-analysis, Systematic review, Ethiopia

## Abstract

**Background:**

Social anxiety disorder is defined as the fear of social situations, incorporating situations that involve contact with strangers. People highly fear embarrassing themselves which includes situations like social gatherings, oral presentations, and meeting new people. People with social phobia have nonspecific fears of practicing vague or, performing specific tasks like eating or speaking in front of others. In people with social anxiety disorder, worry can arise from both the circumstance itself and embarrassment from others, for students, social phobia is an overwhelming fear of speaking in front of others or giving presentations in class. The prevalence of social phobia among different studies in Ethiopia was inconsistent and inconclusive therefore, this study showed the cumulative burden of social phobia among students in Ethiopia.

**Method:**

Observational studies published on social phobia and associated factors among students in Ethiopia were included in this study based on the criteria after independent selection by two authors. Data were extracted by Microsoft Excel spreadsheet to be exported to Stata version 11 for further analysis. The random-effect model was used to estimate the pooled effect size of social phobia and its effect on the previous studies with 95% confidence intervals. Funnel plots analysis and Egger regression tests were conducted to detect the presence of publication bias. Sub-group analysis and sensitivity analysis were done.

**Result:**

A total of 2878 study participants from seven studies were included in this meta-analysis and systematic review. The pooled prevalence of social phobia among students in Ethiopia was 26.81% with a 95% CI (22.31–31.30). The pooled effect size of social phobia in Oromia, Amhara, and SNNPs regions was 24.76%, 24.76%, and 29.47%, respectively. According to the subgroup analysis, university, and college/high school students were 28.05% and 25.34% respectively. Being female [AOR = 2.11 (95% CI 1.72–2.60)], having poor social support [AOR = 2.38 (95% CI 1.54–3.70)], substance use [AOR = 2.25 (95% CI 1.54–3.30)], single parent [AOR = 5.18 (95% CI 3.30–8.12)], and rural residence [AOR = 2.29 (95% CI 1.91–2.75)] were significantly associated in this meta-analysis in Ethiopia.

**Conclusion:**

The pooled prevalence of social phobia in this meta-analysis and systematic review was high (26.81%) among students therefore, the educational bureau needs to work on decreasing the burden of social phobia to raise the academic achievement and creativity of the students. In therapeutic advice like exposure to presentations, family members take the responsibility for the students’ therapy and expose them to various social interactions.

**Supplementary Information:**

The online version contains supplementary material available at 10.1186/s13643-023-02208-2.

## Introductions

According to the Diagnostic and Statistical Manual Fifth Edition (DSM-V), social anxiety is defined as fear or anxiety in social situations, particularly when one is exposed to scrutiny or the possibility of receiving a negative evaluation from others [1]. Social Anxiety Disorder (SAD) is also defined as a marked and constant fear of one or more social and performance situations in which the individual is exposed to unfamiliar people [2]. Students are worried or develop anxiety symptoms due to the unpleasant effect of humiliation by others [3]. This anxious condition hurts the individual and inhibits them from actively engaging in their social surroundings and affects interpersonal relations [4]. The individual fears that he or she will be embarrassed, humiliated, or negatively evaluated by other people around them which can cause to prevent them from academic performance [5].

World Health Organization (WHO) declared social phobia is one of the mental health disorders with major problems that cause disability throughout the world [6]. Social anxiety disorder or social phobia is the most frequent anxiety disorder which causes disability among adolescents [7] and the second most common of all mental disorders next to substance use disorders [8]. Social anxiety disorder is a disabling mental health disorder it accounts 12–16% of the general population [4, 9, 10]. SAD is the second most prevalent anxiety disorder in teenagers and it manifests in early adolescence with early onset [11]. The overall lifetime prevalence of anxiety disorders is 24.9%, from this social phobia accounts a burden of 13.3%, according to the National Comorbidity Survey [12].

Social phobia has a great impact or significant impairment on the young generation of people that need attention for a better life in public society including, students [13]. Anxiety disorders including social phobia are one of the prevalent psychiatric illnesses with a lifetime prevalence of around 17% [14]. Worldwide evidence indicates the lifetime and current prevalence of social anxiety disorder were estimated at 4% and 1.3% respectively [15]. The prevalence of social phobia among students in Parakou, Saudi, and Iran were 11.6%, 59.5%, and 28.6% respectively [16, 17]. The burden of social phobia among high school, college, and university students in Ethiopia was 16.432.8% [2, 18–20].

Social phobia is a condition of intense fear triggered and aggravated by different social situations, in which the students could be judged negatively by other individuals [21]. An extreme fear of being viewed by strangers is a key aspect of social anxiety disorder, and it negatively affects a student's performance in class [22]. Studies have shown that exams, presentations, language, parental rage, criticism in public, overzealous protection, mistreatment, and family provocation were factors of SAD among students [23, 24]. Social fearfulness made people have bad images of their performance in social situations [25]. The determinant factors associated with social phobia were being female, the use of the substance, poor social support, having single parents, having years of study, and having rural residence [2, 20, 26].

The burden of social phobia is varied from place to place because of different perceptions while the children were growing up related to the Evil eye which finally causes SAD in Ethiopia. Social phobia among students is a great problem because of its impact on academic achievements since it prohibited their presentations and speaking in front of teachers and other students due to fear of negative evaluations. Social phobia among high school and university students in Ethiopia is a major burden due to the impact of the habit of not growing interactively socially due to the traditional impact of Evil eyes or Buda. There are a lot of studies conducted in our country with a great variety in the prevalence therefore, this meta-analysis and systematic review provided the pooled prevalence among high school, college, and university students in Ethiopia. Even though many studies were conducted in Ethiopia, the prevalence showed high inconsistency and inconclusive result; therefore, this study provided a pooled impact of social phobia among students in Ethiopia.

## Method and materials

### Study design

Meta-analysis and systematic review were employed according to Preferred Reporting Items for Systematic Review and Meta-Analysis (PRISMA-P 2020) standard [27] (Supplementary material 3).

### Searching strategy

This study was conducted to estimate the pooled prevalence of social phobia and its associated factors among students in Ethiopia. A search of published articles was found by using Pub-Med, Scopus, Cochrane, EMBASE, African journal online (AJOL), HINARI, Science direct, and other gray literature was conducted by searching Google, Google Scholar, and other internet search engines to search any additional articles until the beginning of February 3/2022. The searching terms by the prevalence of social phobia and associated factors OR other Medical Subject Heading (Mesh), keywords, and free text search terms were used. To identify the available pieces of literature, we include different terms for social phobia and combined terms by using Boolean operators searching terms. The querying terms used in these studies (“Social phobia disorders” OR “Performance anxiety disorders”) AND (“associated factors” OR “determinants” OR “students” OR “Ethiopia”). This study focused on the effect of the pooled magnitude of social phobia and its associated factors in Ethiopia.

### Eligibility criteria

This meta-analysis and systematic review collected all peer-reviewed journal articles that addressed social anxiety disorder and/or related factors among Ethiopian students. All observational studies conducted by using different study designs cross-sectional reported from November 2012. Up to February 3, 2022 were included. This systematic review and meta-analysis included publications with full-text and readily accessible, written in English, investigations conducted on humans, and studies of social phobia that were published in peer-reviewed journals. Fortunately, unpublished gray literature was not available after extensive searching on social phobia among students in Ethiopia. Studies found without an abstract, which have no full text, and studies that were difficult to get the required data from studies of social phobia and associated factors in Ethiopia were excluded. Besides this article missing editorial reports, letters, reviews, and commentaries were excluded from the study.

### Measurement of social phobia

Estimating social phobia in Ethiopia was the outcome variable of this meta-analysis and systematic review. Typically, Social Phobia Inventory is used to assess social anxiety (SPIN with a 17-item self-rating scale developed to assess social phobia). This instrument evaluated the validity and reliability of psychometric features in screening social phobia in students and adolescents, including the symptoms domains of fear, avoidance, and physiological arousal. Study participants were asked to rate symptom occurrences as 0 (not at all), 1 (a little bit), 2 (somewhat), 3 (very much), or 4 (extremely during the past week) and the sum score ranged from 0 to 68. A student with a score of 20 and above on SPIN was considered as having social phobia [28, 29].

### Data extraction

Two reviewers (TS and GN) independently identify the papers by their titles and abstracts by the selection criteria. The relevant information was extracted using a Microsoft Excel Spreadsheet after the papers were scrutinized for their titles, abstracts, and entire texts. All studies approved by both reviewers were included and the disagreement was solved by the discussion to get a common consensus. After the agreement, the identified articles the principal investigator’s name, years of publications, study period, study population, and sample size were extracted. The 95% confidence intervals for the combined estimated effect of social phobia and related covariates were also extracted.

### Quality appraisal of the selected literature

To independently evaluate the quality of the articles included in this meta-analysis and systematic review, two authors (TS and GN) by using the standard tool. The Joanna Briggs Institute (JBI) was utilized to evaluate the study quality [30]. Quality measurement instruments were developed for articles that reported prevalence data and observational research. The evaluation of the quality of the included article is the same since the study design for all is the same cross-sectional. The final rating scale of five and above out of nine scores was included in this meta-analysis and systematic review. The discrepancy between reviewers at the time of quality assessment was solved in discussion with a third author to have a common agreement.

### Data processing and analysis

Microsoft Excel was used to construct the extracted data, which was then exported to STATA version-11 statistical software for additional analysis. The estimated pooled effect size and it effect size of all articles with their 95% confidence interval were done by using a random-effect model of meta-analysis. To show the graphic summary of data was determined by the forest plots to estimate the pooled effect size and weight of each recruited study with 95% CI. The degree of heterogeneity among the included articles was done by the index of heterogeneity (*I* ^2^ statistics) [31]. Sub-group analysis by using study area, study design, and study population) and sensitivity analysis was assessed to check the presence of any source of heterogeneity. Publication bias was detected by using Funnel plots analysis and Egger weighted regression tests. A *p* value of < 0.05 in Egger’s test was considered to have statistically significant publication bias [32, 33].

## Results

### Study selection process

**Fig. 1:**
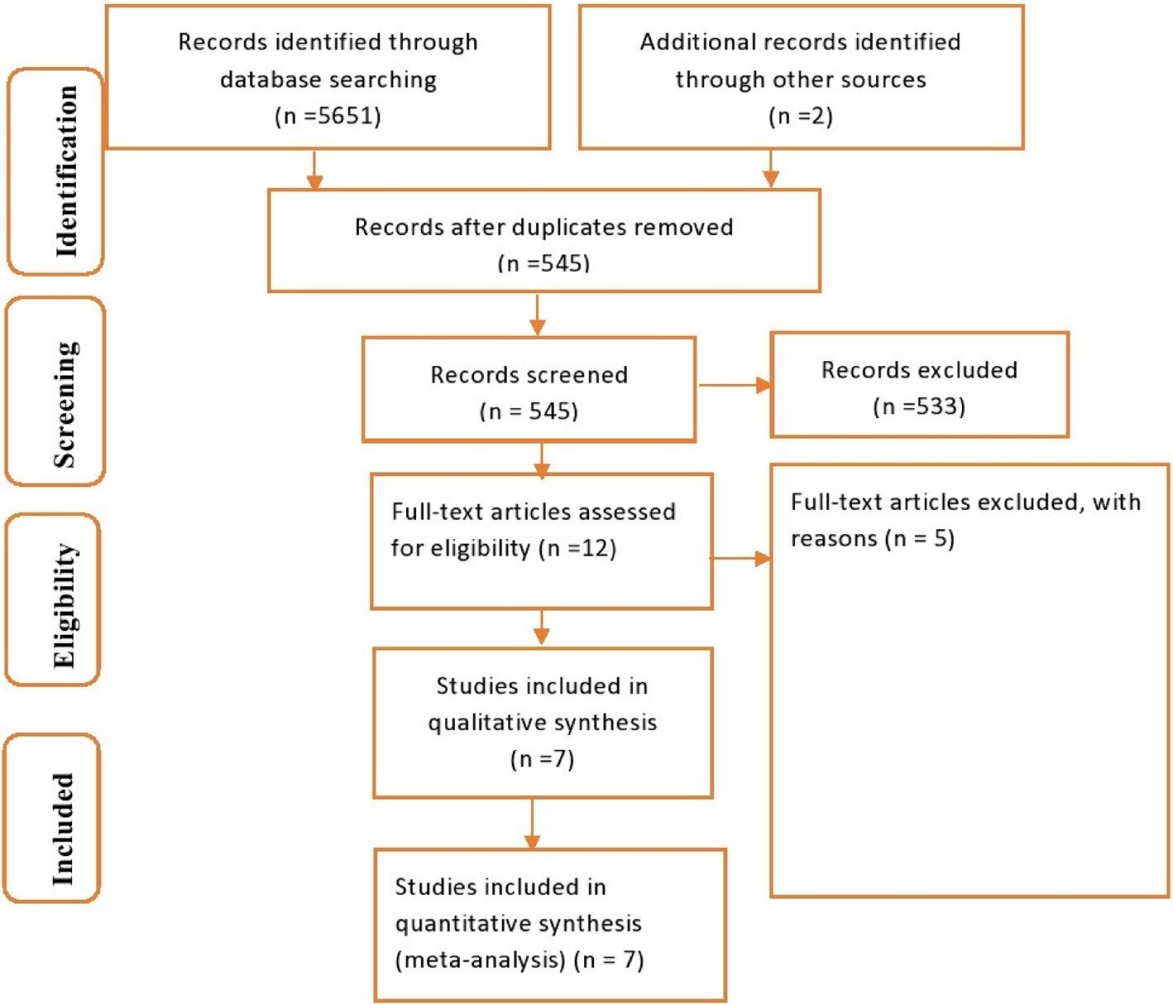
Flow chart describing the selection of studies conducted on social phobia in Ethiopia

There were 5651 studies identified by finding in the search engine and another two studies were added through the additional records. 5106 articles were removed initially after the duplications and 533 studies were excluded by the difference in the study setting and conducted out of Ethiopia. Another 10 papers were excluded after full-text assessment, finally, seven studies were included in this systematic review and meta-analysis. All the papers which fulfilled the inclusion criteria conducted on social phobia among students in Ethiopia were included. Other articles conducted in other settings/other than students, conducted in other countries/other than Ethiopia and papers without the full text were excluded from the study (Fig. 1).

### Characteristics of included studies

A total of seven studies conducted on social phobia and its associated factors among students in Ethiopia were included in this meta-analysis and systematic review. Of these three articles were conducted in Oromia, two were in Amhara, and the remaining two were conducted in the SNNPs region. Four articles from seven included papers were conducted on university students and the rest three articles were on college and high school students. Five of the included articles were conducted before 2020 (Table 1).

**Table 1:** Characteristics of studies included in this meta-analysis among students on social phobia and associated factors in Ethiopia

**Authors**	**Years of publications**	**University**	**Sample size**	**Prevalence of SP in %**
**Desalegn et al. **[18]	2019	Gondar	503	31.2
**Defaru Desalegn et al. **[34]	2021	Wollega	423	32.1
**Reta et al. **[20]	2020	Hawassa	304	32.8
**Hajure et al. **[26]	2020	Mettu	336	16.4
**Shikuro et al. **[2]	2020	Hossahina	403	22.13
**Mekuria et al. **[35]	2017	Woldiya	386	27.5
**Hajure and Abdu **[36]	2020	Mettu	523	26

### The pooled prevalence of social phobia in Ethiopia

**Fig. 2:**
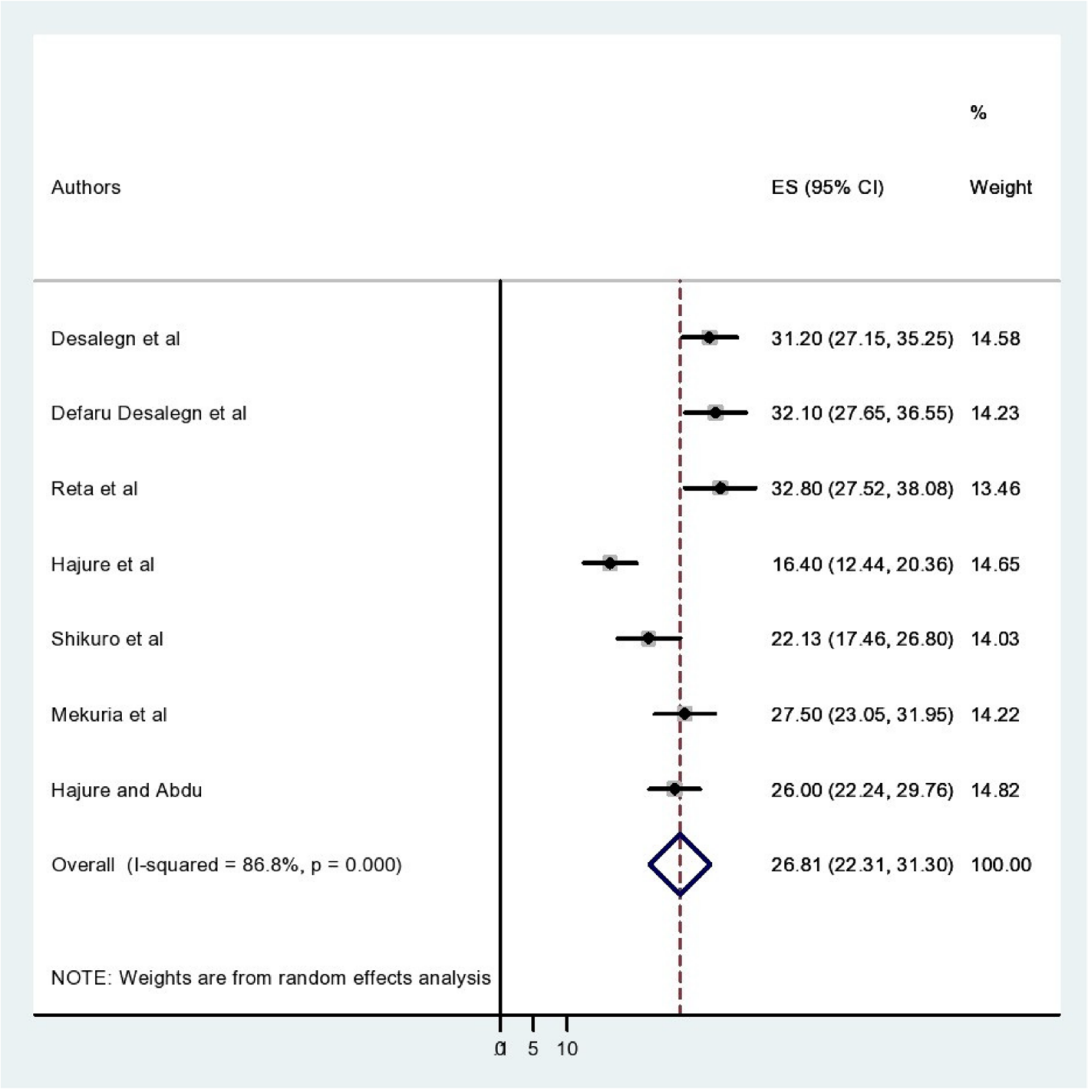
Forest plots that show the pooled level of social phobia among students in Ethiopia

The pooled prevalence of social phobia among students in Ethiopia was 26.81% with a 95% CI (22.31–31.30) (Fig. 2). In this meta-analysis and systematic review study, the combined prevalence of social anxiety disorder among the Oromia, Amhara, and SNNPs regions was 24.76%, 24.76%, and 29.47% respectively. According to subgroup analysis, the burden of social phobia among participants from university students was 28.05% and among college/high school students was 25.34%.

### Heterogeneity and publication bias

**Fig. 3:**
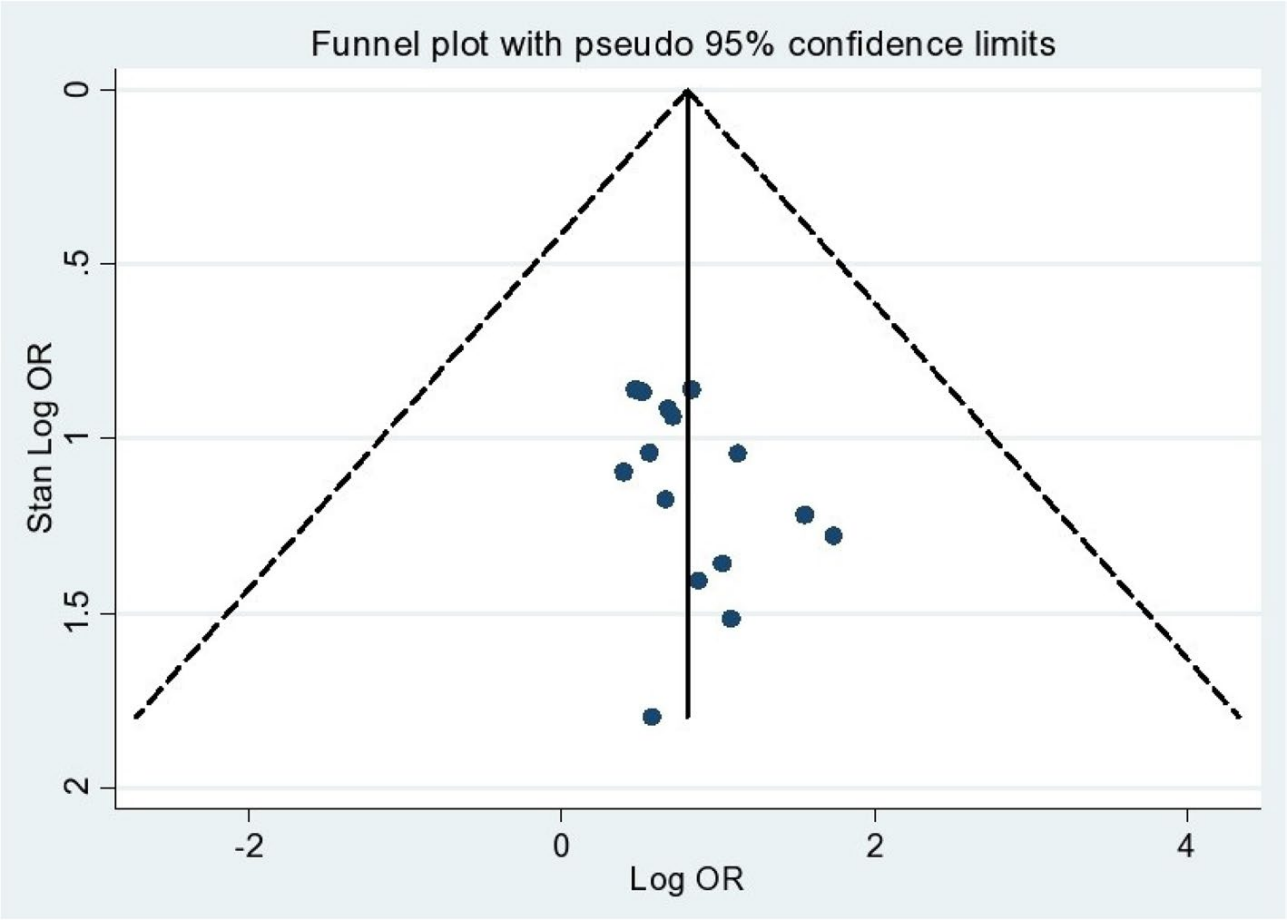
Funnel plot of social phobia among students in Ethiopia

In this meta-analysis and systematic review, heterogeneity was detected with an I^2^ of 98.2% and a *p* value ≤ 0.00. A funnel plot was used to check the existence of potential publication bias in the included articles. The funnel plot falls inside the triangle that indicates the symmetric distribution told indicating the absence of publication bias within the included studies (Fig. 3). Another indicator of the absence of publication bias was Egger’s regression test (*P* = 0.085) (Table 2).

**Table 2:** Egger’s test of social phobia among students in Ethiopia

Std_Eff	Coef	Std. err	*t*	*P* > *t*	[95% Conf	Interval]
slope	.0890368	.3945266	0.23	0.825	− .7571387	.9352122
bias	.6674024	.3597004	1.86	0.085	− .1040781	1.438883

### Subgroup analysis

As previously discussed, the presence of heterogeneity was confirmed, as a result, subgroup analysis was conducted based on region, level of education, and years of publications. The pooled level of social phobia in this study from the incorporated articles in the Oromia region was 24.76%, in the Amhara region, it was 29.47% and in SNNPs 27.39%. The finding of this sub-group analysis indicates the participant with high educational level/university students (28.05%) had higher than others as compared to high school students (25.34%). Therefore, this result showed there is high heterogeneity among some subgroups analysis as indicated by *I* ^2^ which was decreased up to 29 and 31 in some groups which indicate low heterogeneity among the group. The *I* ^2^ shows 88 and 92 which indicate high heterogeneity that enables us to conduct the sensitivity test (*P* ≤ 0.001) (Table 3).

**Table 3:** Subgroup analysis of social phobia and associated factors among students in Ethiopia

Variables	Subgroup	Number of studies	Sample size	Prevalence (95% CI)	*I* ^2^ (%)	*P* value
Region	Oromia	3	1282	24.79(16.07–33.52)	92.8	≤ 0.001
	Amhara	2	889	29.42(25.85–33.09)	31.1	0.228
	SNNPs	2	707	27.39(16.94–37.85)	88.7	0.003
Level of educations	University level	4	1566	28.05(19.91–38.20)	92.7	≤ 0.001
	College/high school	3	1312	25.34(22.40–28.27)	29.5	0.242
Years of publication	≥ 2020	5	1989	25.79(19.77–31.81)	89.5	≤ 0.001
	< 2020	2	889	29.42(25.85–33.09)	31.1	0.228

### A leave-out-one sensitivity analysis

The sensitivity analysis was done to check the heterogeneity of those studies by omitting one author or one study step by step to check the effect of each study on the overall prevalence of social phobia in this systematic analysis. As the result evidenced all the values are within the estimated 95% CI, which indicates the omission of a single study had no significant difference in the prevalence of this meta-analysis (Table 4).

**Table 4:** Sensitivity analysis of social phobia among students conducted in Ethiopia

Study omitted	Estimate 95% CI	Heterogeneity	
		*I* ^2^ (%)	*P* value
Desalegn et al	26.06(21.07–31.05)	87.2	< 0.001
Defaru Desalegn et al	25.93(21.07–30.78)	86.9	< 0.001
Yared Reta et al	25.87(21.08–3067)	87.2	< 0.001
Hajure et al	28.56(25.36–31.75)	68.4	0.007
Shikuro et al	27.58(22.54–32.61)	88.0	< 0.001
Mekuria et al	26.70(21.42–31.98)	88.9	< 0.001
Hajure and Abdu	26.96(21.51–32.41)	89.0	< 0.001

### Associated factors of social phobia

There are associated factors with social phobia among students in this meta-analysis and systematic review in Ethiopia. Among those factors being female was associated with the six previous studies included in this review therefore, it is 2.11 times more likely to have social phobia than other male participants in this study [AOR = 2.11 (95% CI 1.72–2.60)].

Poor social support was linked in three studies on prior research on social phobia among Ethiopian students. The odds of developing social phobia are 2.38 times more likely than other participants who had moderate and high social support [AOR = 2.38 (95% CI 1.54–3.70)].

The other factor associated with social phobia in the three previous studies included in this meta-analysis was substance use. Substance users are 2.25 times riskier to have social phobia among students as compared to non-users [AOR = 2.25 (95% CI 1.54–3.30)].

Participants who have single parents were associated with three previous studies conducted on social phobia which were included in these studies. In this review, the respondent who has single parents is 5.18 times more exposed to social phobia than others [AOR = 5.18 (95% CI 3.30–8.12)].

**Fig. 4:**
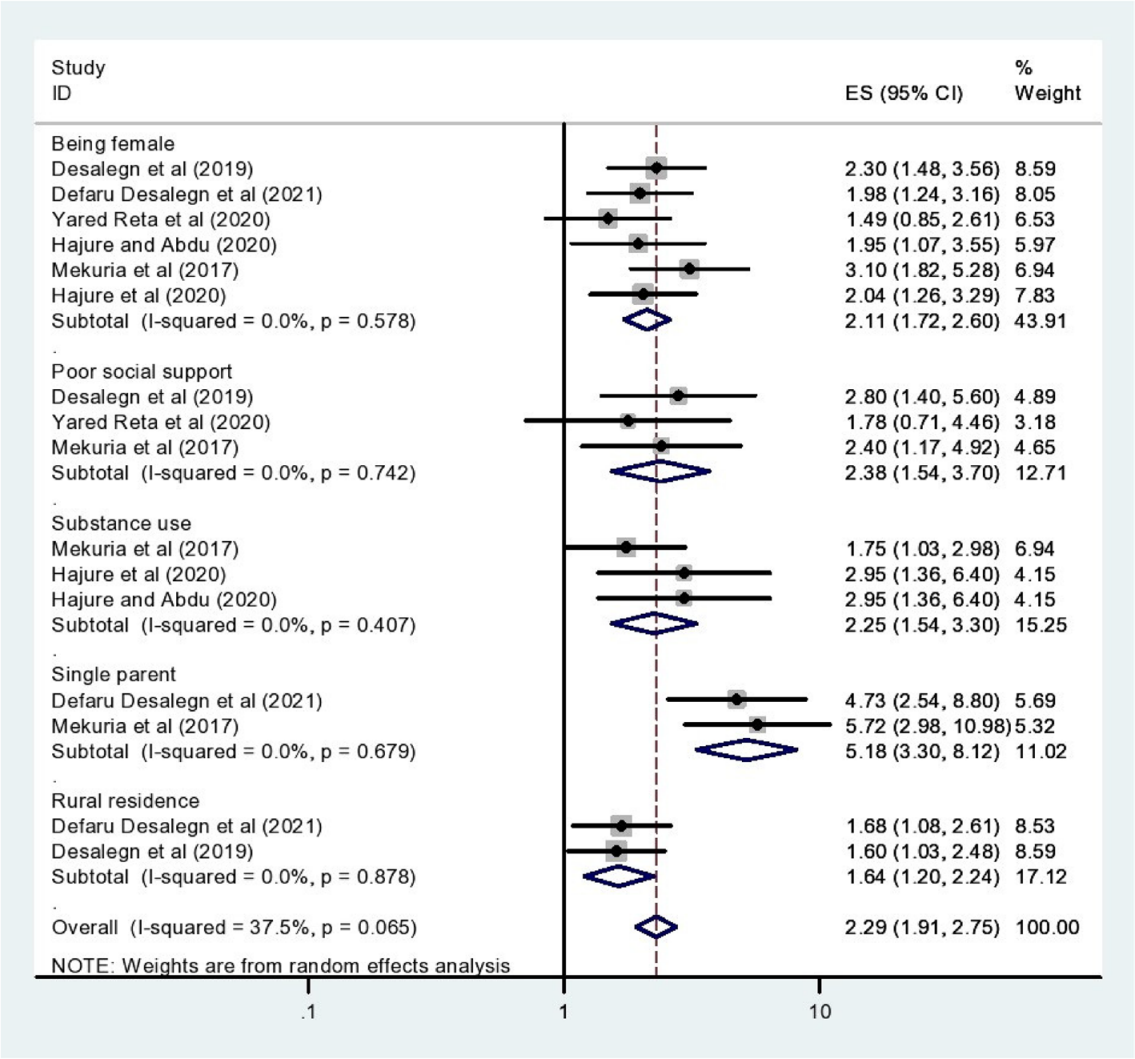
The Forest plot showing different associated factors of social phobia among students in Ethiopia

Other two factors associated with social phobia in Ethiopia among participants from rural residences. The odds of rural residents are 2.29 times more likely to develop social phobia than other urban residents [AOR = 2.29 (95% CI 1.91–2.75)] (Fig. 4).

## Discussion

Social anxiety disorder is an intense, persistent fear or anxiety of being observed and criticized by others which can affect students on their academic performance. The main objective of this meta-analysis was to estimate the pooled effect size of social phobia among students in Ethiopia. The pooled prevalence of this meta-analysis and systematic review was 26.81% with a 95% CI (22.31–31.30). This result was in line with other studies conducted in Nigeria [37], Australia 30.0% [38], and Saudi Arabia 29.8% [39].

This finding is also lower than other studies conducted in Egypt 56.4% [40]. This gap may be caused by the fact that the result in this study is the combined effect size of several studies carried out in Ethiopia, although some of those studies had quite different results from ours. In Ethiopia, there are unique traditional festivals celebrated with high involvement of youths that could have an impact to increase social interaction skills [40].

The result of this study is higher than other studies conducted in Canada 16.3% [41]. This discrepancy may be the result of the fact that Ethiopian children and adolescents receive very little exposure to social interaction and make different decisions than their American counterparts [41]. Despite Ethiopia having great levels of social functioning, the involvement of children does not reveal the fear of social phobia in the long run.

Being a woman was a significant predictor of social anxiety among students related to other characteristics. This finding is in line with other studies conducted in Egypt [19, 42] and Malaysia [43]. The association could be explained by the gender roles that women are expected to play at home, including childrearing and helping with housework [19]. The other reason might be because of the presence of cultural and religious effects on females not equally participating in different tasks [44]. Because of the internalized effect of shame and taking medication for their difficulties, females are more likely to acquire social phobia [42]. This association of females is also evidenced by the physiological effect and the sex role in social and interpersonal relationships [43].

The other determinant factor associated with social phobia in this meta-analysis was participant who have single parents than both parents. This finding is consistent with other studies conducted in [16, 45], and [34]. The association may be due to a lack of family interaction and the emotional toll that losing or separating from parents may have had on the family [45]. The other evidence might be due to the effect of the absence of social interaction, socio-economic, and psychological support which is exposed to social phobia [34, 46].

The other factor associated with social phobia in this systematic review and meta-analysis was substance abuse. This result was in concordance with other studies conducted in the USA [47, 48], and another meta-analysis study [49]. The probable reason for the association could be explained by the reinforcement effect of substances on social anxiety disorder [47]. The other evidence for the association might be the effect of the use of the substance as self-medication for their fear and negative evaluation by others [48, 49].

Poor social support was one of the extra characteristics associated with social anxiety in this study as compared to other study participants who have high social support. This result is in line with other studies conducted at Parakou University [16], and Iran [50]. The possible reason could be the protective effect of being in a comfortable environment and their growth effect [16]. High social connections may also boost confidence and outlook for social abilities, which would be another possible indicator of the association [50].

Participants from the rural residence were associated with social phobia in different studies likewise it is also one of the determinate factors in this meta-analysis in Ethiopia. This result is in line with other studies conducted in India [51] and Saudi Arabia [24]. This could be a result of the rural environment's impact on participants' physical symptoms and their annoyance with novelty, both of which heighten social anxiety symptoms [51]. The possible reason could be the effect of people in the urban area having a high tolerance rate and higher harm avoidance than rural residents [15]. The person from a rural area may have traveled to a modernized location for schooling, which caused them to encounter a new environment and experience problems adjusting to stations [34].

## Limitations of the study

Although this meta-analysis has a great advantage, to give the pooled effect of social phobia among students it has its limitations. As a weakness, the studies included in this review were cross-sectional studies since there is no study on this particular topic and area conducted with other study designs. The small number of articles included in this systematic review and meta-analysis.

## Conclusion and recommendation

The pooled prevalence of social phobia among students in Ethiopia was high. Factors being females, substance users, single parents, participants with poor social support, and rural residents were determinants which is needed to be decreased. An intervention is needed to be taken for the betterment of their life and to increase their educational and innovation skill for students. The health bureau and educational bureau had got their commendation to improve the health status of the students and for better improvement of the educational methodology. The current best education methodology is active learning or student-centered therefore; social phobia needs to be treated first to encourage this kind of educational system. The family members need to take an assignment to follow, increase social interactions, and on the prevention strategies needed for their children.

## Supplementary Information

**Additional file 1. Table S3.** Quality score.

**Additional file 2. **PRISMA checklist social phobia.

**Additional file 3. **

## Data Availability

All the data is present in the manuscript.

## Abbreviations

AOR: Adjusted odd ratio
CI: Confidence interval
DSM: Diagnostic and Statistical Manual of Mental Disorder
SAD: Social anxiety disorder
ICD: International Classification of Disease
SNNP: South Nation Nationality and Peoples
WHO: World Health Organization
