# Supplementary material for: Social phobia of Ethiopian students: meta-analysis and systematic review
Source: Syst Rev. 2023 Mar 14;12:41. doi: 10.1186/s13643-023-02208-2 (PMC10012574; doi:10.1186/s13643-023-02208-2)
Supplement: Supplementary file 1 — Additional file 1. Table S3. Quality score. [file 13643_2023_2208_MOESM1_ESM.docx]

Table_S3 Quality assessment of social phobia among students in Ethiopia included in this meta-analysis and systematic review.

| Author, year of  Publication | Q1 | Q2 | Q3 | Q4 | Q5 | Q6 | Q7 | Q8 | Q9 | Total score (9%) |
| --- | --- | --- | --- | --- | --- | --- | --- | --- | --- | --- |
| Desalegn et al,2019 | Y | Y | NA | Y | Y | Y | Y | Y | Y | 8 |
| Hajure et.al,2020 | Y | Y | Y | Y | Y | Y | Y | Y | Y | 9 |
| Reta et.al,2020 | Y | Y | Y | Y | Y | Y | Y | Y | Y | 9 |
| Defaru Desalegn et al,2021 | Y | Y | Y | Y | NA | Y | Y | NA | Y | 7 |
| Mekuria et.al,2017 | Y | Y | Y | Y | Y | Y | Y | Y | Y | 9 |
| Shikuro et.al,2020 | Y | NA | Y | Y | Y | Y | Y | Y | NR | 7 |
| Hajure and Abdu et.al,2020 | Y | Y | Y | Y | Y | Y | Y | Y | Y | 9 |

**Key:** **Y**= Yes; **NR**= Not reported, **NA**=Not appropriate

**Question codes:**

1. Was the sample frame appropriate to address the target population?

2. Were study participants sampled in an appropriate way?

3. Was the sample size adequate?

4. Were the study subjects and the setting described in detail?

5. Was the data analysis conducted with sufficient coverage of the identified sample?

6. Were valid methods used for the identification of the condition?

7. Was the condition measured in a standard, reliable way for all participants?

8. Was there appropriate statistical analysis?

9. was the response rate adequate, and if not, was the low response rate managed appropriately?
